# V-ATPase modulates exocytosis in neuroendocrine cells through the activation of the ARNO-Arf6-PLD pathway and the synthesis of phosphatidic acid

**DOI:** 10.3389/fmolb.2023.1163545

**Published:** 2023-04-07

**Authors:** Qili Wang, Alexander Wolf, Sebahat Ozkan, Ludovic Richert, Yves Mely, Sylvette Chasserot-Golaz, Stéphane Ory, Stéphane Gasman, Nicolas Vitale

**Affiliations:** ^1^ Institut des Neurosciences Cellulaires et Intégratives, CNRS UPR 3212 and Université de Strasbourg, Strasbourg, France; ^2^ Laboratoire de Bioimagerie et Pathologies, Faculté de Pharmacie, CNRS UMR and Université de Strasbourg, Strasbourg, France

**Keywords:** chromaffin cells, V-ATPase, phospholipase D, phospholipids, neurosecretion, membrane fusion

## Abstract

Although there is mounting evidence indicating that lipids serve crucial functions in cells and are implicated in a growing number of human diseases, their precise roles remain largely unknown. This is particularly true in the case of neurosecretion, where fusion with the plasma membrane of specific membrane organelles is essential. Yet, little attention has been given to the role of lipids. Recent groundbreaking research has emphasized the critical role of lipid localization at exocytotic sites and validated the essentiality of fusogenic lipids, such as phospholipase D (PLD)-generated phosphatidic acid (PA), during membrane fusion. Nevertheless, the regulatory mechanisms synchronizing the synthesis of these key lipids and neurosecretion remain poorly understood. The vacuolar ATPase (V-ATPase) has been involved both in vesicle neurotransmitter loading and in vesicle fusion. Thus, it represents an ideal candidate to regulate the fusogenic status of secretory vesicles according to their replenishment state. Indeed, the cytosolic V1 and vesicular membrane-associated V0 subdomains of V-ATPase were shown to dissociate during the stimulation of neurosecretory cells. This allows the subunits of the vesicular V0 to interact with different proteins of the secretory machinery. Here, we show that V0a1 interacts with the Arf nucleotide-binding site opener (ARNO) and promotes the activation of the Arf6 GTPase during the exocytosis in neuroendocrine cells. When the interaction between V0a1 and ARNO was disrupted, it resulted in the inhibition of PLD activation, synthesis of phosphatidic acid during exocytosis, and changes in the timing of fusion events. These findings indicate that the separation of V1 from V0 could function as a signal to initiate the ARNO-Arf6-PLD1 pathway and facilitate the production of phosphatidic acid, which is essential for effective exocytosis in neuroendocrine cells.

## Introduction

Neurons and neuroendocrine cells release neurotransmitters, neuropeptides, and hormones by the calcium-regulated exocytosis of synaptic vesicles and large dense core vesicles, respectively. This process can be divided into multiple steps, including vesicle biogenesis, maturation, and transport of vesicles, followed by their docking at the exocytotic site and priming. Vesicles fully acquire the ability to fuse and release their contents in response to Ca^2+^ influx (Tanguy et al., 2016). Ca^2+^ entry through voltage-gated channels is detected by Ca^2+^ sensor proteins, which ultimately triggers the membrane fusion machinery (Rizo, 2018). A transient fusion pore connecting the vesicle lumen to the extracellular space opens and allows the diffusion of neurotransmitters and hormones outside cells. Based on the “kiss-and-run” model, this pore can either rapidly close after releasing small portions of the vesicular content or expand to allow either partial or full vesicle collapse into the plasma membrane, leading to three different modes of release (Yu et al., 2018). Ultimately, partially or fully empty secretory vesicles are retrieved by endocytosis and recycled for a novel round of secretion after replenishment.

SNARE proteins are widely involved in vesicular transport across eukaryotes. In the case of neurosecretion, the classical SNARE complex comprises the v-SNARE synaptobrevin (VAMP2), which is located in the vesicle membrane, and the t-SNAREs syntaxin 1 and SNAP-25, which are mainly present in the plasma membrane (Sutton et al., 1998). It is widely accepted that SNAREs, aided by the calcium sensor synaptotagmin, can directly facilitate lipid bilayer fusion. The exact nature of the fusion pore is still a matter of debate, and it appears that different modes of transmitter release may arise from differences in the organization of the membrane fusion machinery. Therefore, SNARE assembly may generate the mechanical force required to mix lipids or, alternatively, bring membranes together and provide a framework for downstream proteins that create a channel-like pore or catalyze changes in lipid pore intermediates (Jahn and Südhof, 1999; Lauwers et al., 2016).

V-ATPases are large multimeric enzymes found in many intracellular membrane compartments, including synaptic vesicles and secretory granules, where they generate vesicular proton gradients and membrane potential (Forgac, 2007). Acidification of these organelles is required for many cellular processes (e.g., maturation or degradation of proteins, receptor-mediated endocytosis, and proton-coupled transport of small molecules) (Forgac, 2007). This gradient and potential are also needed for neurotransmitter uptake by selective transporters in synaptic vesicles and secretory granules (Moriyama et al., 1992).

V-ATPases are organized into two domains, V1 and V0. The cytosolic V1 domain contains eight different subunits (A–H) with subunit A responsible for ATP hydrolysis, thereby providing the energy for the V0 membrane domain to translocate protons. V0 contains a, c, d, and e subunits associated with five c subunits (Forgac, 2007). V-ATPase is expressed in all eukaryotic cells with the primary function of proton pumping. This activity can be controlled in multiple ways, including subcellular targeting of different isoforms, modulating the expression level or stability of different subunits, regulating the coupling efficiency of ATP-hydrolysis to proton transport, and modulating the reversible association of V0 and V1 subdomains (Maxson and Grinstein, 2014).

In addition to its role in proton translocation, V0 has more recently been linked to intracellular membrane fusion (Peters et al., 2001; Peri and Nüsslein-Volhard, 2008; Strasser et al., 2010; Williamson et al., 2010), neurotransmitter release (Hiesinger et al., 2005; Bodzęta et al., 2017), and exocytosis (Liégeois et al., 2006; Poëa-Guyon et al., 2013), suggesting that it is directly involved in the fusion of two membrane compartments. It was initially proposed that V0 could serve as a component of the fusion pore (Morel et al., 2001; Peters et al., 2001) or promote lipid mixing and the creation of a lipidic fusion pore (Strasser et al., 2010; El Far and Seagar, 2011). V0 has indeed been shown to interact with SNARE proteins (Galli et al., 1996; Peters et al., 2001; Morel et al., 2003; Hiesinger et al., 2005; Di Giovanni et al., 2010). Furthermore, V0 could act as a pH sensor (Hurtado-Lorenzo et al., 2006; Hosokawa et al., 2013) and facilitate the priming steps necessary to prepare mature secretory vesicles for exocytosis (Morel et al., 2003). Previous studies rely on the long-term genetic invalidation of specific subunits. However, this approach does not rule out indirect effects of pH gradient alteration in membrane fusion deficits. The use of chromophore-assisted light inactivation techniques, which consist of inserting a small tetracystein motif allowing for the specific inactivation of V0 or V1 subunits upon light illumination, has recently validated the notion that photo-inactivation of the V0c or V0a subunits leads to a rapid impairment of synaptic transmission in neurons and of catecholamine release in chromaffin cells (Poëa-Guyon et al., 2013; Rama et al., 2019).

Although the original idea that V0 could form the fusion pore between secretory vesicles and the plasma membrane has been recently ruled out (Morel et al., 2014; Morel and Poëa-Guyon, 2015), the exact mode of action by which V-ATPase regulates exocytosis remains elusive. Interestingly, a direct interaction between V0c and VAMP2 has been observed in mammalian neurons, establishing a first link with SNARE-dependent exocytosis. A second interesting lead comes from a fascinating study, revealing an interaction between V-ATPase and the pair Arf6/ARNO on endosomes (Hurtado-Lorenzo et al., 2006). Arf6 is a small GTPase mainly involved in vesicular trafficking and signaling lipid synthesis, whereas ARNO is a guanine exchange factor (GEF) for Arf proteins including Arf6. As Arf6 and ARNO were shown to be essential regulators of exocytosis through the activation of phospholipase D1 (PLD1) and the synthesis of phosphatidic acid near the exocytotic sites (Galas et al., 1997; Caumont et al., 1998; Yang and Mueckler, 1999; Caumont et al., 2000; Vitale et al., 2002a; Vitale et al., 2002b; Matsukawa et al., 2003; Liu et al., 2005; Béglé et al., 2009; Pelletán et al., 2015), we decided to explore the possibility that V-ATPase could modulate this pathway. Here, we report that V0a1 interacts with ARNO after the stimulation of neuroendocrine cells. The overexpression of a GFP-tagged N-terminal V0 peptide competing with the endogenous interaction between V0 and ARNO prevented both Arf6 and PLD activation and strongly reduced exocytosis recorded from cells by carbon fiber amperometry. Interestingly, these inhibitory effects on the kinetic parameters of individual fusion events are very similar to what was found after PLD inhibition, supporting the notion of an interplay between V0 and the ARNO/Arf6/PLD pathway in regulated exocytosis.

## Results

### ARNO and V0a interact during exocytosis in bovine chromaffin cells

**FIGURE 1:**
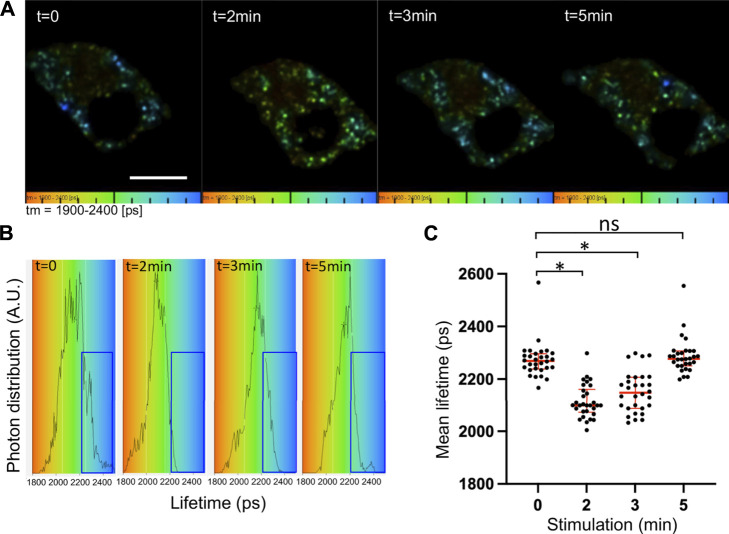
Increased V0a1–ARNO interaction after chromaffin cell stimulation. Chromaffin cells expressing V0a1-NowGFP and ARNO-shadowG were stimulated at different times with 10 µM of nicotine. Fluorescence decays of V0a1-NowGFP were recorded by fluorescence lifetime imaging microscopy (FLIM) and analyzed with a one-component exponential function. **(A)** Representative lifetime color-coded images of granular V0a1-NowGFP from two cells at different times after stimulation (Bar = 10 µm). Fluorescence lifetime values of V0a1-NowGFP were represented using a color code scale ranging from 1900 ps (orange) to 2400 ps (blue). Dots correspond to V0a1-NowGFP-containing granules. **(B)** V0a1-NowGFP lifetime distribution for different times after stimulation from cells recorded in **(A)**. Blue boxes highlight a decrease in the longer lifetime values after cell stimulation. **(C)** Plot of individual mean lifetime values obtained from *n* = 30 cells in three independent preparations. Medians with an interquartile range are illustrated in red. *p < 0.05 compared to stimulation time 0.

Both co-immunoprecipitation and pull-down experiments have revealed an interaction of ARNO with the V0a2 subunit of the V-ATPase along the endolysosomal pathway in epithelial cells (Hurtado-Lorenzo et al., 2006). In agreement with these observations, we consistently found that endogenous V0a1 co-precipitated with endogenous ARNO from the chromaffin cells extract (data not shown). After cell stimulation, there was a tendency for an increase in the co-immunoprecipitation of V0a1 and ARNO, which, however, did not reach statistical significance. One possible reason is that the V0a1–ARNO interaction is transient during exocytosis and may not be easily captured by an immunoprecipitation approach. We, thus, used a Förster resonance energy transfer (FRET) approach and fluorescence lifetime imaging microscopy (FLIM) to further investigate the interaction between ARNO and V0a1, a subunit of V0 associated with secretory granules in chromaffin cells (Figure 1). When FRET occurs, the non-radiative energy transfer between the donor and the acceptor leads to donor emission quenching and a decrease in the fluorescence lifetime of the donor. Compared to the classical intensity-based FRET approaches, FLIM–FRET has many advantages as 1) lifetime decay does not require calibration and is not sensitive to inner filter effects, photobleaching, and possible spectral crosstalk between the acceptor and donor; 2) it is internally calibrated and thus independent of the donor and acceptor concentrations; and 3) it enables the identification of fractions of molecules involved in FRET. However, since a minimal number of photons (∼10^4^) need to be collected for a good signal-to-noise ratio, acquisition times in the order of one minute are needed with our conventional laser scanning FLIM setup. To improve the FRET dynamic range but also to reduce spectral contamination and provide more stable and sensitive measurements, we used NowGFP as the donor and the dark GFP ShadowG mutant as the acceptor.

FLIM measurements of V0a1-NowGFP in the whole chromaffin cell revealed significant changes in the lifetime of NowGFP after the stimulation of exocytosis (Figures 1A, B). The average lifetime dropped significantly from 2272 ± 41 ps before stimulation to 2113 ± 50 ps and 2150 ± 63 ps after 2 min and 3 min of stimulation, respectively. Notably, after 5 min of stimulation, the average lifetime was back to normal levels at 2287 ± 42 ps (Figure 1C). Interestingly, a close examination of individual lifetime distribution profiles revealed an increase in the distribution between 1800 and 2000 ps at the expense of the distribution observed between 2200 and 2500 ps (Figure 1B, blue box). These observations suggest that a subpopulation of V0a1-NowGFP is probably getting near ARNO-ShadowG after cell stimulation. Our experimental conditions did not allow the precise measurement of V0a1 signals on individual secretory granules since our FRET–FLIM measurements took around 60 s, during which time most secretory granules showed significant motion. Quantitative analysis indicated that individual cells did not respond with similar kinetics, but that on average, the most important drop in lifetime occurred after 2 min of stimulation and that the average lifetime was close to the control non-stimulated condition, 5 min after stimulation (Figure 1). These observations suggest that a significant increase in the interaction between ARNO and V0a occurs after the stimulation of exocytosis.

### Preventing V0a–ARNO interaction abolishes Arf6 activation during exocytosis in neuroendocrine PC12 cells

**FIGURE 2:**
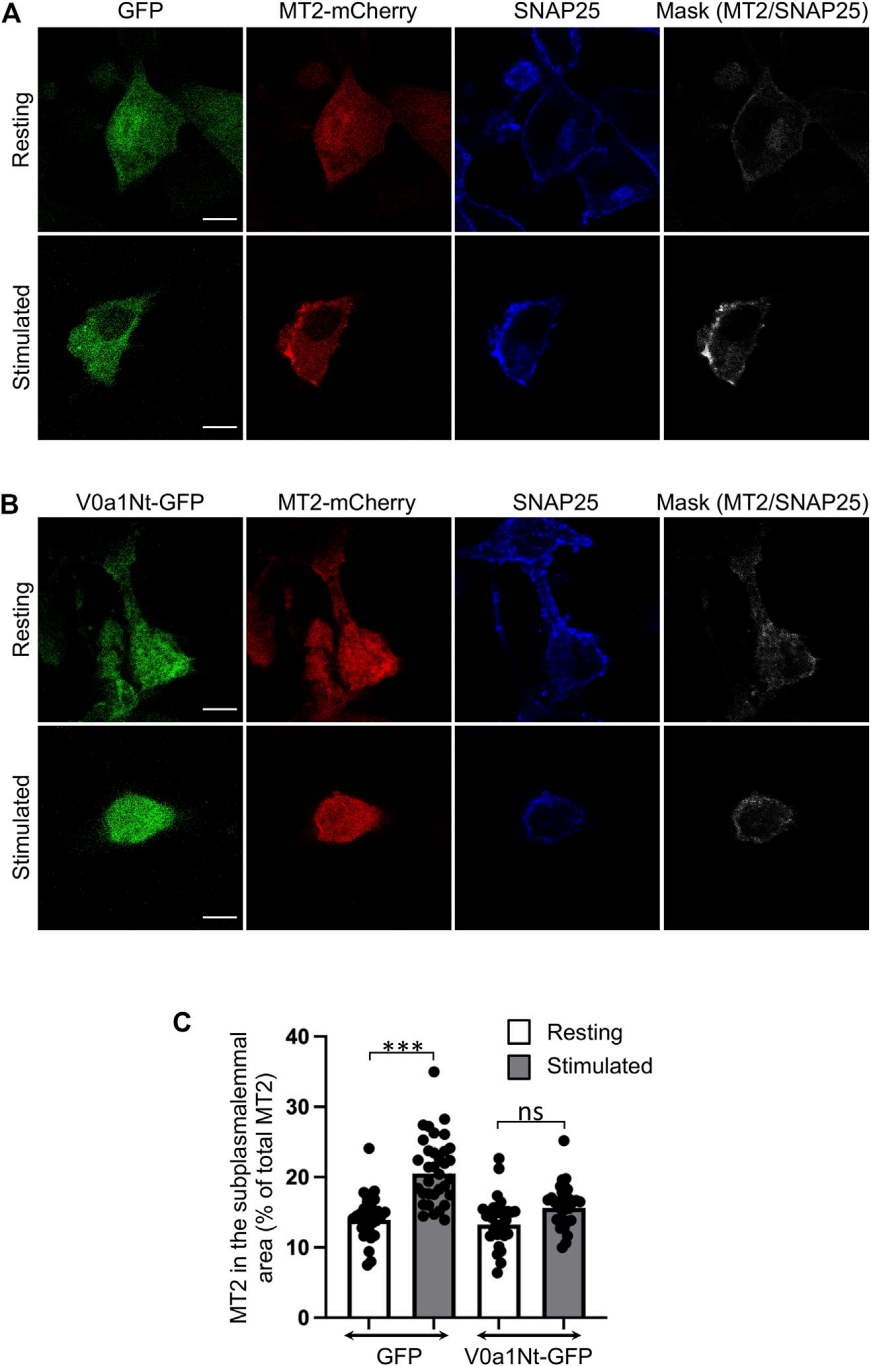
Interfering with V0a1–ARNO interaction reduces Arf6 activation after PC12 cell stimulation. Distribution of the MT2-mCherry probe in PC12 cells co-expressing GFP **(A)** or V0a1Nt-GFP **(B)** in resting condition or after a 10-min stimulation with a [59 mM] K^+^ solution. After fixation, cells were immunostained for SNAP25. Masks indicate the co-localization between MT2-mCherry and SNAP25 signals. Bar = 5 µm. **(C)** Levels of MT2-mCherry in the subplasmalemmal area were measured and presented (*n* > 30 cells for each condition from three independent experiments). ***p < 0.001 compared to resting condition.

We previously reported that Arf6, a key regulator of PLD1 for exocytosis (Vitale et al., 2002a), is activated by the GEF ARNO (Béglé et al., 2009). The overexpression of the amino-terminal domain of V0a1 was previously shown to prevent the GDP/GTP exchange activity of ARNO on Arf6 *in vitro* (Hurtado-Lorenzo et al., 2006). We combined this approach by expressing this short competing peptide (MGELFRSEEMTLAQLFLQS) fused to GFP (V0a1Nt-GFP) together with the expression of MT2-mCherry, a reporter for GTP-loaded active Arf6 in cells (Béglé et al., 2009; Tanguy et al., 2019). Even though we tried to co-express the two plasmids that are necessary for this experiment in bovine chromaffin cells, we were not able to get enough co-transfected cells to perform the proper analysis. However, by using this approach on PC12 cells, a cell line isolated from a chromaffin cell tumor, we found that Arf6 is activated at the plasma membrane when exocytosis is triggered with a depolarizing potassium solution in agreement with previous observations (Béglé et al., 2009). This effect is severely impaired in cells expressing V0a1Nt-GFP. Indeed, in control cells expressing GFP, an increase in MT2-mCherry recruitment to the cell periphery was observed upon stimulation, as revealed by an increase in the colocalization with the plasma membrane marker SNAP-25 (Figure 2A). This plasma membrane recruitment of MT2 in stimulated cells is largely abolished in cells expressing V0a1Nt-GFP (Figure 2B). The quantification of the level of MT2-mCherry in the subplasmalemmal area validated these observations (Figure 2C). These data suggest that V0a–ARNO interaction may be essential for the proper activation of Arf6 during regulated exocytosis. In other words, V0a1 could be a regulator of the GEF activity of ARNO toward Arf6 during exocytosis.

### Interfering with the V0a–ARNO interaction abolishes PLD activation during exocytosis in PC12 cells

**FIGURE 3:**
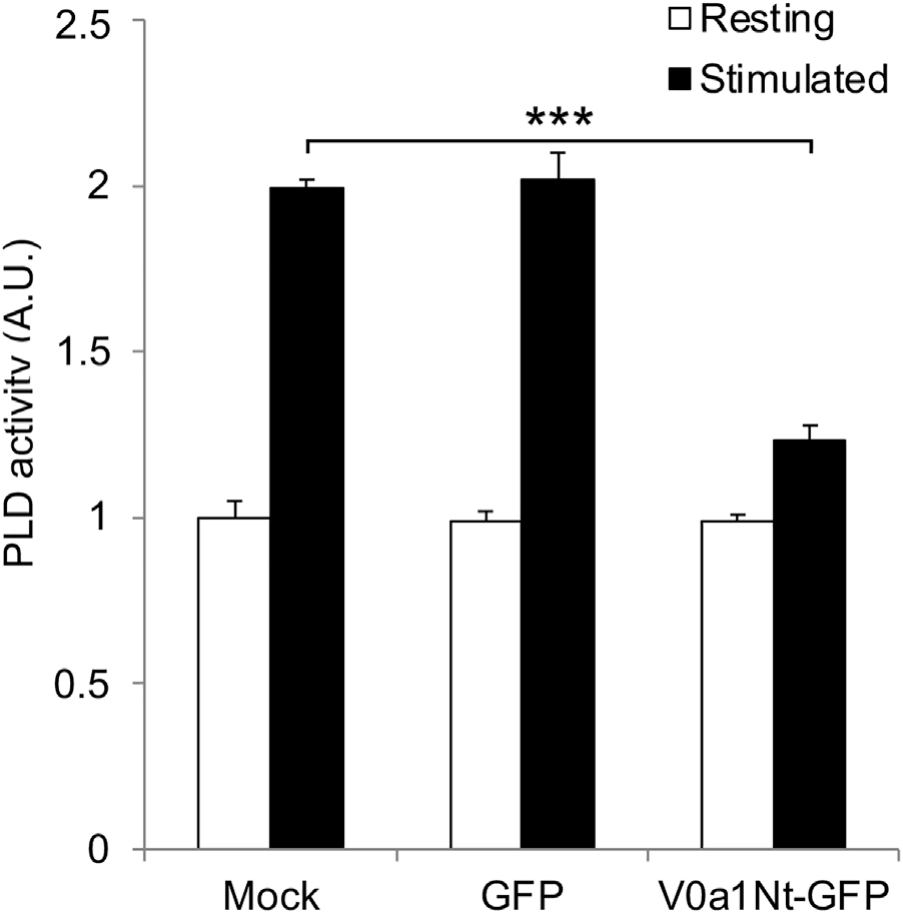
Interfering with V0a1–ARNO interaction reduces PLD activation after PC12 cell stimulation. Mock-transfected cells or cells expressing GFP or V0a1Nt-GFP were kept in Locke’s solution (resting) or stimulated with a [59 mM] K^+^ solution for 10 min (stimulated). Cells were lysed on ice, and lysates were used to measure the PLD activity. Data are expressed as the mean values ± SD from independent experiments (*n* = 3), each obtained from quadruplicates in individual experiments. ***p < 0.001 compared to control (Mock).

An increase in PLD activity during exocytosis was reported in different cellular models, including neuroendocrine chromaffin and PC12 cells, and appears to predominantly depend on PLD1 activation (Caumont et al., 1998; Vitale et al., 2001). Several regulators of PLD1 during exocytosis have been identified, including the kinase Rsk2 (Zeniou-Meyer et al., 2008) and several small GTPases, including RalA, Rac1, and Arf6 (Vitale et al., 2005; Bader and Vitale, 2009; Béglé et al., 2009; Momboisse et al., 2009). Accordingly, PLD activity measured from the PC12 cell extracts nearly doubled after exocytosis stimulation with a depolarizing potassium solution in control mock-transfected cells or in cells expressing GFP alone (Figure 3). Strikingly, the overexpression of V0a1Nt-GFP strongly prevented this stimulation-dependent increase in PLD activity (Figure 3), suggesting that V0a–ARNO interaction may be essential for the optimal stimulation of PLD during exocytosis. These data also reveal that the Arf6 activation pathway represents a major contributor of PLD activity required for exocytosis.

### V0a–ARNO association is essential for PA synthesis at the PC12 cell periphery during exocytosis

**FIGURE 4:**
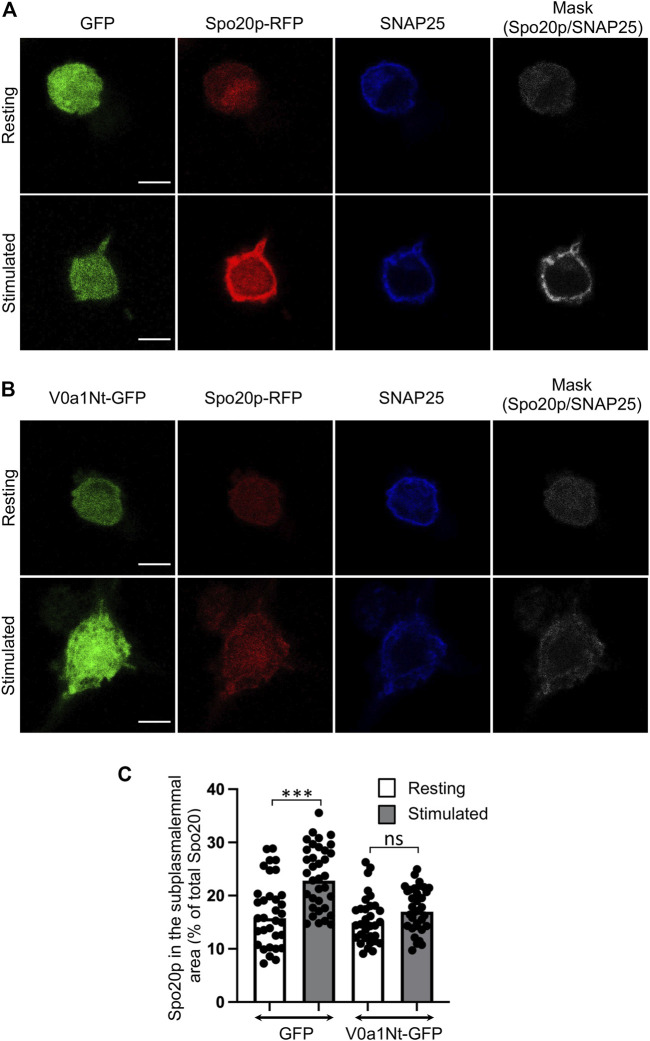
Interfering with V0a1–ARNO interaction reduces PA synthesis at the plasma membrane after PC12 cell stimulation. Distribution of Spo20p–RFP probe in cells co-expressing GFP **(A)** or V0a1Nt-GFP **(B)** in resting condition or after a 10-min stimulation with a [59 mM] K^+^ solution. After fixation, cells were immunostained for SNAP25. Masks indicate the co-localization between Spo20–RFP and SNAP25 signals. Bars = 5 µm. **(C)** Levels of Spo20–RFP in the subplasmalemmal area were measured and presented (*n* > 30 cells for each condition from three independent experiments). ***p < 0.001 compared to resting condition.

Increased levels of PA, the product of PLD activity, were reported in various secreting cells. The PA-binding domain of the yeast protein Spo20p fused to RFP (Spo20p–RFP) acts as a PA sensor and has been particularly useful in visualizing this increase of PA at the plasma membrane (Kassas et al., 2012; Kassas et al., 2017). Further morphometric electronic microscopy analysis revealed that this PA sensor was found to be enriched in portions of the plasma membrane from chromaffin cells where a secretory granule appears morphologically docked, suggesting that PA is synthesized near exocytotic sites (Zeniou-Meyer et al., 2007). Accordingly, we found, in control cells expressing GFP, that Spo20–RFP was recruited to the subplasmalemmal area of PC12 cells following stimulation, as revealed by an increase of colocalization with the plasma membrane marker SNAP-25 (Figure 4A). This recruitment of Spo20p–RFP in stimulated cells is largely abolished in cells expressing V0a1Nt-GFP (Figure 4B). The quantification of the level of Spo20p–RFP in the subplasmalemmal area validated these observations (Figure 4C). These data suggest that the V0a–ARNO interaction is also critical for the synthesis of PA at the plasma membrane during exocytosis.

### Preventing V0a–ARNO interaction affects exocytosis in neuroendocrine chromaffin cells

**FIGURE 5:**
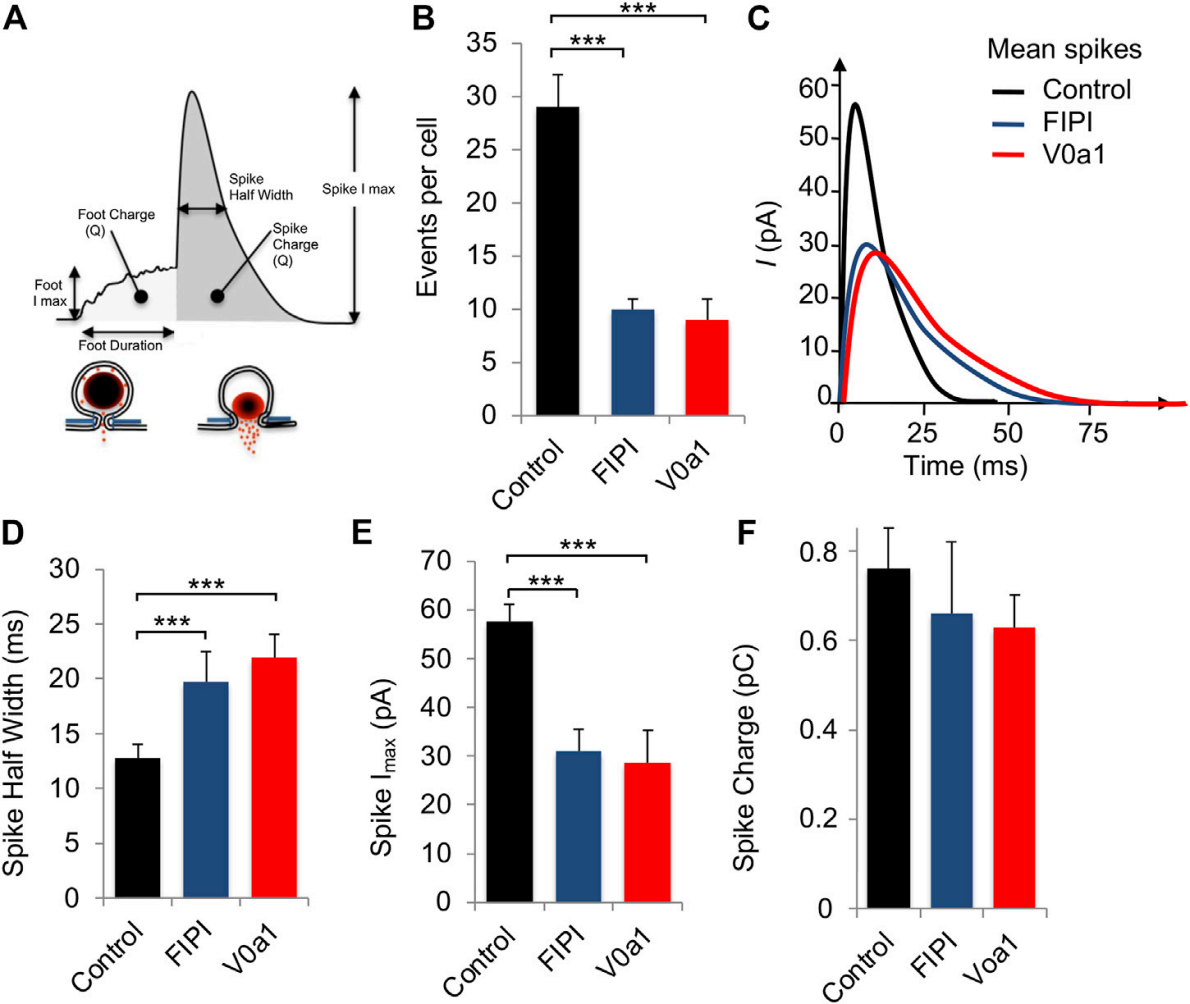
Interfering with V0a1–ARNO interaction affects individual spike kinetic parameters from chromaffin cells. **(A)** Scheme showing the different parameters of amperometric spikes that were analyzed. **(B–E)** Chromaffin cells in culture expressing GFP (control) or V0a1Nt-GFP (V0a1) or untransfected cells treated with 750 nM of FIPI for 1 h were stimulated with a local application of 100 µM of nicotine for 10 s and catecholamine secretion was monitored using carbon-fiber amperometry. **(B)** Histogram illustrates the number of amperometric spikes recorded per cell. **(C)** Average spike profile for cells expressing GFP (Control) or V0a1Nt-GFP (V0a1) or treated with FIPI (FIPI). Parameters of individual spikes including spike half width **(D)**, spike amplitude **(E)**, and spike charge **(F)** are expressed as the mean ± S.D. (*n* > 75 cells for each condition from three independent cell cultures). ***p < 0.001 compared to control.

**FIGURE 6:**
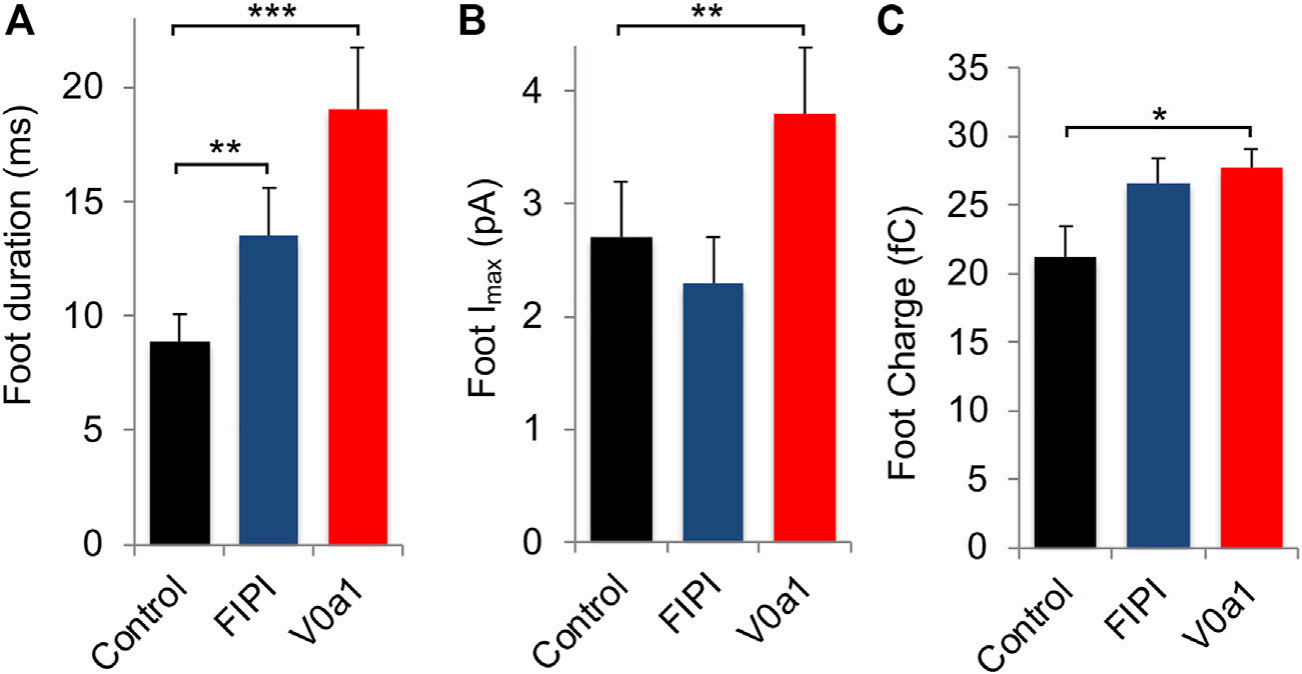
Interfering with V0a1–ARNO interaction affects pre-spike foot kinetic parameters from chromaffin cells. Individual pre-spike parameters from cells recorded in Figure 5 include pre-spike duration **(A)**, pre-spike amplitude **(B)**, and charge **(C)**. Data are expressed as the mean ± S.D. (*n* > 75 cells for each condition from three independent cell cultures). *p < 0.05, **p < 0.01, and ***p < 0.001 compared to control.

Carbon-fiber amperometry is an elegant and powerful approach for evaluating kinetic parameters of exocytosis from single cells secreting catecholamines, as it allows the measurement of various parameters of individual secretion events (Figure 5A). A typical amperometric profile shows successive spikes, each corresponding to the detection of single secretory granule fusion events. Interestingly, expression of V0a1Nt-GFP (V0a1) or inhibiting PLD activity in chromaffin cells by using FIPI, a dual PLD1/2 inhibitor FIPI, had similar effects on different parameters. On the one hand, those two treatments reduced the number of spikes per cell compared to cells expressing GFP alone (Figure 5B). On the other hand, they also modified individual spike parameters (Figure 5C) by increasing the spike half-width (Figure 5D) and decreasing their amplitude (Figure 5E). In contrast, no significant effect on spike charge has been observed (Figure 5F). Therefore, when PLD activation is impaired, either by directly inhibiting this enzyme or by interfering with the ARNO-Arf6-PLD pathway, 1) the total number of exocytic events is reduced, 2) the kinetics of individual fusion events are slowed, and 3) the total amount of catecholamine released by the event is not modified. More precisely, an increase in the time to peak (rise time) observed upon V0a1Nt-GFP expression most likely represents a slowing down in the speed of the fusion pore expansion. Amperometric spikes are often preceded by small increases in the current amplitude called pre-spike feet, which are believed to represent the detection of small amounts of catecholamine leaking through the initial fusion pore (Tryoen-Tóth et al., 2013). The expression of V0a1Nt-GFP significantly increased the amplitude and duration of pre-spike feet, indicating that the V0a–ARNO interaction affects the pore size and stability (Figure 6). Interestingly, very similar changes in these pre-spike parameters were also observed when chromaffin cells were treated with FIPI (Figure 6), highlighting the link between V0 and PLD activation during the initial stages of membrane fusion. Altogether, at a physiological level, hampering with PLD activation would thus reduce the number of granules releasing their content without modifying the quantum of release itself, but affecting the kinetics of release. These effects will most likely impact the ability to respond to a stressful situation.

## Discussion

In neurosecretory cells, the production of specific lipids at and near exocytotic sites appears to be an important regulatory element of the fusogenic status of secretory vesicles (Gasman and Vitale, 2017; Tanguy et al., 2020). Although the pathway involving the production of PA through PLD activation by Arf6 was shown to be an important trigger for exocytosis in different cell types (Galas et al., 1997; Caumont et al., 1998; Yang and Mueckler, 1999; Caumont et al., 2000; Vitale et al., 2002a; Vitale et al., 2002b; Matsukawa et al., 2003; Liu et al., 2005), the exact signal that engages this pathway has remained elusive. ARNO, an Arf6 GEF, activates Arf6 in different membrane trafficking processes (Hernández-Deviez et al., 2004; Eva et al., 2012; Zhu et al., 2017), but again, the activation mechanism of ARNO was never clearly identified. The observation that different subunits of the V-ATPase, a known participant in exocytosis (Galli et al., 1996; Morel et al., 2001; Peters et al., 2001; Morel et al., 2003; Hiesinger et al., 2005; Liégeois et al., 2006; Peri and Nüsslein-Volhard, 2008; Di Giovanni et al., 2010; Strasser et al., 2010; Williamson et al., 2010; El Far and Seagar, 2011; Poëa-Guyon et al., 2013; Bodzęta et al., 2017; Lévêque et al., 2023), could interact with ARNO/Arf6 on early endosomes to regulate the endocytic degradative pathway in epithelial cells (Hurtado-Lorenzo et al., 2006) prompted us to probe the possible involvement of the V-ATPase in the regulation of the ARNO-Arf6-PLD pathway in neuroendocrine cells.

The c subunit of V0 was shown to specifically interact with Arf6 in epithelial cells, but the functional consequences of this interaction were not investigated, and the domains involved in both proteins were not identified (Hurtado-Lorenzo et al., 2006). The same study, further established by co-immunoprecipitation and pull-down experiments, that the a-subunit of V0 directly interacts with ARNO on early endosomes (Hurtado-Lorenzo et al., 2006). In this case, the amino-terminal domain of V0a is involved in the interaction with ARNO and the expression of the V0a-Nt peptide fused with GFP was reported to act as a competitor (Hurtado-Lorenzo et al., 2006). Finally, this interaction was reported to promote the GEF activity of ARNO and therefore led to Arf6 activation (Hurtado-Lorenzo et al., 2006). Using a FLIM–FRET-based approach, here, we found that V0a1 expressed on chromaffin granules interacts with ARNO within 2 min of cell stimulation, suggesting that V0a1 could be an activator of the ARNO-Arf6-PLD pathway during exocytosis. It is worth noting that this timing is consistent with a previous study reporting on FRET that the highest Arf6 activation was detected nearly 2 min after exocytosis stimulation in PC12 cells (Béglé et al., 2009).

We found that the overexpression of V0a1Nt-GFP in PC12 cells inhibited the activation of Arf6, as detected by reduced recruitment of the active Arf6 sensor MT2. It also decreased the stimulation of PLD activity and prevented the PA synthesis at the plasma membrane, as visualized by lower recruitment of the PA probe Spo20p–RFP. One possible interpretation of this observation is that V0a1 interaction with plasma membrane-associated ARNO occurs after granule tethering/docking at the exocytotic site. The association of Arf6 with V0c that we also detected by co-immunoprecipitation in PC12 cells (data not shown) may also contribute to the close contact between V-ATPase, ARNO, and Arf6. The subsequent activation of Arf6 leading to PLD activation and PA synthesis might, therefore, also favor granule docking and ultimately fusion.

Preventing V0a1–ARNO interaction dramatically reduced the number of single exocytosis events and affected their kinetics in chromaffin cells, as reported by carbon-fiber amperometry recordings, highlighting the importance of this interaction for granule docking and fusion. Overall, the profile of the remaining single spikes was smaller and broader, in agreement with a delay in the expansion of the fusion pore when V0a1-Nt-GFP is expressed. In line with this model, the duration of the pre-spike foot was found to be increased, indicating greater fusion pore stability. Importantly, similar observations were also found in cells treated with the PLD inhibitor FIPI, suggesting that a major outcome of the V0a1-Nt-GFP expression is reminiscent of a downstream alteration of PA synthesis. It is, however, also worth noting that V0a1-Nt-GFP expression affected pre-spike foot amplitude, an effect not seen after PLD inhibition, raising the possibility that other targets of the V-ATPase might also be affected. Among those, the SNARE protein VAMP2, which interacts directly with V0c, is a prominent candidate (Di Giovanni et al., 2010).

We previously reported, using co-immunoprecipitation experiments, that the V0/V1 assembly is dependent on intragranular pH (Poëa-Guyon et al., 2013). In line with our findings, fluorescence recovery after photobleaching (FRAP) of GFP-tagged V-ATPase subunits also indicated that V0/V1 assembly is correlated with intragranular pH, with most acidic synaptic vesicles showing low amounts of V1 (Bodzęta et al., 2017). Furthermore, an optogenetic approach revealed that incompletely filled synaptic vesicles fuse with lower release probability (Herman et al., 2018). We can, therefore, propose that the V0/V1 association–dissociation represents a mechanism allowing the preferential fusion of fully acidified and filled vesicles devoid of V1. This mechanism may, therefore, provide a control step to avoid exocytosis of empty or incompletely filled vesicles. Evidently, the V-ATPase also plays an important role in synaptic vesicle recycling and refilling (Bodzęta et al., 2017). Since most constituents of secretory granules from neuroendocrine cells are also recycled by compensatory endocytosis (Ceridono et al., 2011), the possible involvement of the pathway described here for secretory vesicle recycling remains to be explored.

## Materials and methods

*Antibody and chemicals*—Anti-SNAP-25 was purchased from BioLegend (Cat #: 836303) and Hoechst 33342 from ThermoFisher. The small-molecule dual PLD1/PLD2 potent inhibitor FIPI, derived from halopemide, was purchased from Merck.

*Plasmids*—Oligonucleotides corresponding to the 19 residues of the N-terminal part of V0a1 were annealed and cloned into pEGFP-N3 through BglII and BamHI restriction sites as described (Recchi and Chavrier, 2006). NowGFP (Addgene plasmid #74749) was cloned into pV0a1 IV-EGFP in KpnI and MunI restriction sites, whereas ARNO was cloned into CMV-ShadowG (Addgene plasmid #104620) in BglII and HindIII restriction sites, as described previously (Vitale et al., 1996).

*Chromaffin cell culture and electroporation* ***—*** Freshly dissected primary bovine chromaffin cells were cultured in Dulbecco’s modified Eagle’s medium (DMEM) in the presence of 10% fetal calf serum, 10 µM cytosine arabinoside, 10 µM fluorodeoxyuridine, and antibiotics, as described previously (Vitale et al., 1993). Plasmid expressing GFP-V0a1-Nt was introduced into chromaffin cells (10^7^ cells) by Amaxa Nucleofactor systems (Lonza) according to the manufacturer’s instructions and as described previously (Chasserot-Golaz et al., 2005).

*Carbon-fiber amperometry* ***—*** 48h/72 h after transfection, catecholamine secretion was evoked by applying K^+^ (100 mM) in Locke’s solution without ascorbic acid for 10 s to single cells by means of a glass micropipette positioned at 30–50 μm from the cell. For amperometry recordings, transfected cells, expressing GFP close to the center of the plates, were randomly selected. Electrochemical measurements of catecholamine secretion were performed using 5-µm diameter carbon-fiber electrodes (ALA Scientific) held at a potential of + 650 mV compared with the reference electrode (Ag/AgCl) and approached close to the transfected cells, as described previously (Gabel et al., 2015). To reduce variations due to electrodes, cells from each condition were alternatively recorded every 2–3 cells. Amperometric recordings were performed with an AMU130 (Radiometer Analytical) amplifier, sampled at 5 kHz, and digitally low-pass-filtered at 1 kHz. Analysis of amperometric recordings was performed as described previously (Houy et al., 2015), allowing automatic spike detection and extraction of spike parameters (Gabel et al., 2015; Houy et al., 2015; Tanguy et al., 2020). Spike charge corresponds to the area below the spike curve and was calculated with a macro (obtained from Dr. R. Borges’s laboratory; https://rborges.webs.ull.es/), written for Igor software (Wavemetrics). The number of amperometric spikes was counted as the total number of spikes with an amplitude >5 pA.

*PC12 cell culture and transfection* ***—*** PC12 cells were grown in DMEM supplemented with glucose (4500 mg/L) and containing 30 mM NaHCO_3_, 5% fetal bovine serum, 10% horse serum, 100 units/mL penicillin/streptomycin, and 100 μg/mL kanamycin, as described previously (Béglé et al., 2009). For immunofluorescence experiments, expression vectors were introduced into PC12 cells using lipofectamin 2000 on adherent cells, according to the manufacturer’s instructions. Under these conditions, the transfection efficiency ranged from 60 to 75%, and the co-transfection rate was greater than 90%.

*Determination of PLD activity* ***—*** 72 h after transfection, PC12 cells were washed four times with Locke’s solution and then incubated for 10 min in a calcium-free Locke’s solution (basal PLD activity) or stimulated in Locke’s solution containing a depolarizing concentration of K^+^ [59 mM]. The medium was then replaced with 100 µL of an ice-cold Tris 50 mM, pH 8.0, solution, and the cells were broken by three freeze-and-thaw cycles. The samples were collected and mixed with an equal amount of the Amplex Red reaction buffer (Amplex Red Phospholipase D assay kit, Molecular Probes), and the PLD activity was estimated after 1 h incubation at 37°C using a Mithras (Berthold) fluorometer. A standard curve was performed with purified PLD from *Streptomyces* chromofuscus (Sigma).

*FRET*–*FLIM imaging—*Chromaffin cells expressing V0a1-NowGFP and ARNO-ShadowG (ShadowG is a mutation of GFP that is used as a dark acceptor in FRET experience) were incubated in Locke’s solution in 8-well Ibidi plates. The cells were then stimulated by the addition of concentrated nicotine solution to reach 20 µM final concentration. The FRET signals were quantified before and during stimulation by FLIM. The FLIM setup was a homemade two-photon excitation scanning microscope based on an Olympus IX70 inverted microscope with a 60 × 1.2 NA water immersion objective and a thermostat at 37°C. Excitation at 930 nm was provided using a broadband femtosecond laser (Insight DeepSee, Spectra Physics). The laser power was adjusted to optimize the parameters of acquisition (best signal-to-noise ratio without the pile-up effect). Fluorescence photons were collected in the descanned fluorescence collection mode using a short-pass filter with a cut-off wavelength of 680 nm (F75-680, AHF filter) and two fiber-coupled APD (SPCM-AQR-14-FC, PerkinElmer) in a single-photon counting mode. The photon signals were collected through a time-correlated single-photon counting (TCSPC) module (SPC830, Becker & Hickl) for FLIM measurements. To obtain appropriate photon counts, FLIM images were acquired for 60 s and analyzed using SPCImage software (v.7.4, Becker & Hickl). A binning of two was applied to the FLIM image to obtain more than 3,000 counts per decay to optimize the fit.

*Immunolabeling and confocal microscopy* ***—*** PC12 cells expressing MT2-mCherry or Spo20–RFP constructs together with GFP or V0a1-Nt-GFP were fixed with 4% paraformaldehyde in a serum-free medium for 10 min at room temperature after stimulation of not more than 10 min with, respectively, a depolarizing K^+^[59 mM] or normal Locke solution. The cells were then permeabilized in PBS containing 4% paraformaldehyde and 0.1% Triton-X100 for 10 min at room temperature. SNAP-25 (1:500 dilution) antibodies were used as markers for the plasma membrane and revealed with a goat-anti-mouse IgG-Alexa 647 (1:1000 dilution, Molecular Probes, Thermo Scientific). The cellular distributions of the MT2-mCherry or Spo20–RFP and markers were examined with a Leica SP5 II confocal microscope equipped with an oil immersion × 63 objective (Plan Apochromat n. a. = 1.4). Digital images were acquired in the equatorial plane. Mask images showing the double-labeled pixels and revealing the areas of co-localization of MT2-mCherry or Spo20–RFP with SNAP-25 were obtained by using the “and” function of the image calculator of FIJI software. The quantification of MT2-mCherry or Spo20–RFP signals at the plasma membrane on randomly selected cells was performed using a homemade macro on Icy software. The cells were delimited and mCherry/RFP fluorescence intensity was measured in the subplasmalemmal area (5 pixels wide) by using the SNAP25 signal. The percentage of each probe in the subplasmalemmal area was then obtained by multiplying the mean fluorescence intensity of the given probe in this compartment by its area and dividing it by the total area of the cell, multiplied by its mean fluorescence intensity.

*Statistical analysis* ***—*** The number of experiments and repeats are indicated in the figure legends. The normality of data distribution was verified, and statistical analysis was performed with t-tests (Figures 2C, Figure 3, Figure 4C) or with the ANOVA test (Figures 1, 5, 6) relative to the indicated control.

## Data Availability

The raw data supporting the conclusion of this article will be made available by the authors, without undue reservation.

## Abbreviations

ARNO, Arf nucleotide-binding site opener; FLIM, fluorescence lifetime imaging microscopy; FRET, Förster resonance energy transfer; GEF, guanine nucleotide exchange factor; PA, phosphatidic acid; PBS, phosphate saline buffer; PLD, phospholipase D; V-ATPase, vacuolar-ATPase.
